# Bibliography

**Published:** 1888-12

**Authors:** 


BIBLIOGRAPHY.

Foster's Illustrated Encyclopædic Medical Dictionary: D. Appleton & Co., 1, 3 and 5 Bond Street, New York. Sold by subscription only. Complete in four volumes. The first volume, consisting of 752 pages, covering the alphabet from *a* to *cacos*, is now ready and before us for review. The amount of labor necessary to have gotten out such a work is simply stupendous. It is an encyclopædic compendium of medical and allied sciences founded upon independent reading, and is not a compilation from other medical dictionaries. It represents the unremitted labors of Dr. Frank P. Foster and twelve collaborators through two years time. Not only is the etymological signification of words given, but a most satisfactory and complete idea of the object for which the words stand is presented to the mind of the reader. The derivation of words is followed out most carefully in all instances, and their foreign equivalents in nearly all cases are given, each word is pronounced, thus meeting a need which we have all felt in the medical dictionaries commonly in use.

The definitions are based upon independent readings of the productions of prominent medical and scientific authors and writers. It is amply and well illustrated with good wood cuts. It is printed on an excellent quality of paper, and the typographical and press work are most excellent, as that in general of D. Appleton & Co.

As examples of the accuracy and extent of the work we will quote one or two examples. "Amalgam, N., a²m-a²l′-ga²m. (The figures refer to letters in foot note; in this case to a, as in at.) Lat., *amal-gama.* From λαγμα, an emollient; (from μάλάσσειν, to make soft.) Fr., *amalgame.* Ger., *Quecksilberlegirung.* It., Sp., *amalgama.* 1. A combination of mercury with some other metal. 2. A soft alloy. 3. In general any mixture of dissimilar things. (B. 2)—*Chasés new a.* A dental a. Made by melting 40 parts of pure silver, and adding 30 pure tin, stirring, and adding 5 each of tears of zinc and antimony and 10 of beeswax, rasping and removing all traces of iron with a magnet. Mercury being finally added at the time it is used. (L. 125)—*Contour a.* A dental a. Consisting of 58 parts of silver, 37 of tin, and 5 of gold, and a sufficient quantity of mercury. (L. 125)—*Dental a.* Fr., *amalgame des dentistes.* Ger., *zahn amalgam.* A composition used for filling cavities in teeth, made of mercury and one or more other metals. Gold, silver, copper, tin and zinc are most commonly used for this purpose etc., including the formulæ for *electrical*, *facing*, *front teeth*, *kein-mayer*, *submarine* and Townsend's amalgams, with directions how to keep, etc.

Under the head of bacteria fourteen pages are given, including the physiological and morphological appearances together with a complete bigliography. An example is found in Miller's *episilon—β.* "Miller's name for a slender, straight, or more or less curved, non-motile rod-form, found in carious teeth along with four other micro-organisms, designated respectfully as α, β, γ and ς. It often occurs in pairs, assuming then an ς or ο-shape. It liquifies gelatine. Applied, to teeth it produccs caries. In its growth it resembles the *spirillum* of Finkler Prior, and is probably identical with it." Under the head of bone ten pages are devoted to the subject with ample illustrations. Too much cannot be said in praise of the work, but we will refrain at the present in order to have something to say of the succeeding volumes.

A Practical Treatise on Artificial Crown and Bridge-work. By George Evans. With 500 illustrations. Octavo, pp. 258 and index. Philadelphia: The S. S. White Dental Manufacturing Co., 1888. Price, cloth, $3.00.

The work is well gotton up and withal makes a very creditable appearance. The illustrations are profuse, many of them original, while others have been taken from the *Cosmos.* The work to a certain extent is a compilation on the subject. The author has done his work well, and has evidenced a power of description in explaining mechanical methods that is possessed by few writers. As a reference handbook the volume deserves high rank and should be in the hands of every student as well as practitioner, for in it may be found all the practical methods that have been presented on the subject of crown and bridge-work up to date of issue.

